# Evaluating DNA Methylation in Random Fine Needle Aspirates from the Breast to Inform Cancer Risk

**DOI:** 10.1155/2022/9533461

**Published:** 2022-08-11

**Authors:** Kala Visvanathan, Ashley Cimino-Mathews, Mary Jo Fackler, Pritesh S. Karia, Christopher J. VandenBussche, Mikiaila Orellana, Betty May, Marissa J. White, Mehran Habibi, Julie Lange, David Euhus, Vered Stearns, John Fetting, Melissa Camp, Lisa Jacobs, Saraswati Sukumar

**Affiliations:** ^1^Johns Hopkins Bloomberg School of Public Health, Baltimore, MD, USA; ^2^Women's Malignancy Program, Johns Hopkins Kimmel Comprehensive Cancer Center, Johns Hopkins School of Medicine, Baltimore, MD, USA; ^3^Department of Pathology, Johns Hopkins School of Medicine, Baltimore, MD, USA; ^4^Department of Surgical Oncology, Johns Hopkins School of Medicine, Baltimore, MD, USA

## Abstract

**Introduction:**

Critical regulatory genes are functionally silenced by DNA hypermethylation in breast cancer and premalignant lesions. The objective of this study was to examine whether DNA methylation assessed in random fine needle aspirates (rFNA) can be used to inform breast cancer risk.

**Methods:**

In 20 women with invasive breast cancer scheduled for surgery at Johns Hopkins Hospital, cumulative methylation status was assessed in a comprehensive manner. rFNA was performed on tumors, adjacent normal tissues, and all remaining quadrants. Pathology review was conducted on blocks from all excised tissue. The cumulative methylation index (CMI) for 12 genes was assessed by a highly sensitive QM-MSP assay in 280 aspirates and tissue from 11 incidental premalignant lesions. Mann–Whitney and Kruskal Wallis tests were used to compare median CMI by patient, location, and tumor characteristics.

**Results:**

The median age of participants was 49 years (interquartile range [IQR]: 44–58). DNA methylation was detectable at high levels in all tumor aspirates (median CMI = 252, IQR: 75–111). Methylation was zero or low in aspirates from adjacent tissue (median CMI = 11, IQR: 0–13), and other quadrants (median CMI = 2, IQR: 1–5). Nineteen incidental lesions were identified in 13 women (4 malignant and 15 premalignant). Median CMI levels were not significantly different in aspirates from quadrants (*p* = 0.43) or adjacent tissue (*p* = 0.93) in which 11 methylated incidental lesions were identified.

**Conclusions:**

The diagnostic accuracy of methylation based on rFNA alone to detect premalignant lesions or at-risk quadrants is poor and therefore should not be used to evaluate cancer risk. A more targeted approach needs to be evaluated.

## 1. Introduction

Current risk assessment tools lack the discriminatory power to accurately determine an individual's breast cancer risk. This limits our ability to personalize screening and prevention strategies [1, 2]. Genes epigenetically silenced by DNA methylation in tumors disrupt multiple normal cellular functions, including cell cycle regulation, cell signaling, cell differentiation, immortalization, and DNA repair [3]. This type of epigenetic reprogramming can occur before the development of pathologically detectable lesions [4, 5]. Risk stratification/early detection of breast cancer could be improved by the incorporation of biomarkers that reflect molecular changes associated with carcinogenesis [6]. Novel approaches, however, are needed to detect these changes.

Random fine needle aspiration (rFNA) of the breast is a relatively noninvasive technique, compared to a core or excisional biopsy. It has been used in clinical trials to assess breast cancer risk and to detect molecular changes due to short term interventions [7–11]. Using rFNA, cytological atypia is associated with increased breast cancer risk [7]. High DNA methylation was also found to be significantly correlated with increasing cytological atypia in pooled rFNA samples obtained in a cross-sectional study of 380 healthy volunteers [10]. Methylated markers associated with breast cancer have also been detected in the contralateral breast of women with invasive tumors using rFNA [8–12]. More recently, a panel of methylation markers measured on FNA samples was shown to successfully differentiate between tumor and benign lesions [12]. The objective of the current study was to determine whether presence of methylation based on analysis of rFNA samples could be used to identify premalignant tissue and inform cancer risk.

## 2. Materials and Methods

### 2.1. Study Population

Eligible participants were women ≥18 years old, with a biopsy-proven diagnosis of invasive breast cancer, a single discrete lesion on a mammogram that was ≥1 cm, no plan to receive neoadjuvant therapy before surgery, and a surgery date for a mastectomy or lumpectomy at the Johns Hopkins Hospital. Participants were ineligible if they had multicentric disease based on imaging, a prior cancer other than basal or squamous carcinoma of the skin and/or cervical carcinoma in situ, a prior unilateral or bilateral prophylactic mastectomy or lumpectomy, had breast implants, were on antibiotic treatment for an infection, or had previously taken tamoxifen, raloxifene, or an aromatase inhibitor for breast cancer prevention. All participants provided written informed consent. The study was approved by the institutional review board at the Johns Hopkins Bloomberg School of Public Health (JHBSPH).

### 2.2. Study Design

The study schema is displayed in Supplementary Figure S1. At enrollment, participants completed a brief questionnaire that included both demographic and risk factor information, and a blood sample was collected. On the day of surgery, after the patient was anesthetized, but prior to the definitive surgical procedure, intraoperative rFNA of 5 unaffected quadrants (3 in the ipsilateral breast and 2 in the contralateral breast, upper inner, and upper outer) was performed by one of 5 participating breast surgeons. An effort was made by the surgeon to target the glandular areas of each quadrant to increase cellular yield based on both mammogram and physical examination. The rFNA procedure itself was performed by deep infiltration into the breast tissue with a 21-gauge needle attached to a syringe. Two-needle punctures were performed per quadrant, with 25 excursions. The sample for each quadrant was pooled and placed into a single tube maintained on ice. The rFNA samples were placed in Cytolyt®, a methanol-based preservative solution.

Post surgery, a cold pack and/or compression bandages were applied to the breast. The excised tissue was immediately transported to the Department of Pathology, as per standard clinical workflow. rFNA of the tumor and adjacent normal tissue was performed by one of 3 participating pathologists using the aforementioned approach for the unaffected tissue. Adjacent normal tissue was defined as grossly normal-appearing mammary parenchyma located within 1 cm of the grossly identifiable tumor mass if present. The rFNA samples were filtered and stored in vials at −80 degrees C freezer in the JHBSPH Core Laboratory. A follow-up call was performed two weeks after the surgery to document any side effects related to the procedure. Adverse effects were graded based on CTCAE classification [13].

#### 2.2.1. Methylation Assay

QM-MSP was performed in rFNA samples to quantitate the level of methylation for the following 12 genes: *RASSF1, RASGRF2, AKR1B1, COL6A2, CCND2, TM6SF1, APC, ZNF671, TMEFF2, HOXB4, RARB,* and *HIST1H3C* [10, 12, 14–17]. All samples from one individual were run in the same batch to minimize interassay variability bias. Individual gene methylation (*M*) was calculated as %*M* = (^#^methylated copies/^#^methylated + ^#^unmethylated copies detected)×100, averaged across duplicates. Cumulative methylation index (CMI) is the sum of the *M* in each of 12 genes. Each batch of samples has a control that is 100% methylated, 100% unmethylated, and water only.

For each tissue sample, four slides of formalin-fixed paraffin-embedded (FFPE) tissues were dewaxed in xylene for 20 minutes at room temperature, and then air-dried. Tissue was scraped into a 500-microliter microcentrifuge tube. DNA was extracted in 50 microliters 10 mM Tris, 150 mM NaCl, 2 mM EDTA, 0.5% SDS, and 100 *μ*g/ml salmon sperm DNA containing 40 micrograms of proteinase K for 16 h at 56°C. The lysate was heat-inactivated for 20 min at 70°C and then, DNA was converted with sodium bisulfite using the EZ DNA Methylation kit (Zymo Research). QM-MSP was performed as previously described [14, 18]. Laboratory personnel was blinded to all clinical information for both FNA and tissue samples.

#### 2.2.2. Pathology Review

The surgically excised specimens were handled as per the clinical standard of care; routine sections of the tumor, adjacent normal, and unaffected quadrants were submitted to FFPE blocks and subsequent histologic examination. After completion of the standard of care diagnostic review, the H&E stained slides from every tissue block (*N* = 103) were retrieved from the pathology archives for study review by the study surgical pathologist (A.C-M). The histologic findings in the slides from the tumor, adjacent normal, and unaffected normal quadrants were recorded.

#### 2.2.3. Statistical Analysis

Mann–Whitney and Kruskal Wallis tests were used to compare median CMI by selected patient and tumor characteristics, and to compare median CMI and gene-specific estimates by location within the ipsilateral and contralateral breast and pathological diagnoses.

Our original sample size was 40 women based on having 90% power to detecting cumulative methylation in rFNA collected from at least 36 tumors with a one-sided error rate of 5%. A planned futility analysis was incorporated apriori into the study design after the enrollment of 20 women to avoid unnecessary rFNA procedures of remaining tissue (adjacent normal or other quadrants). The plan was to stop performing rFNAs if we are unable to detect any methylation in the remaining tissue of at least 10/20 women. Recruitment for the study was stopped after the enrollment of 20 women due to low levels of methylation in the remaining tissue.

## 3. Results

### 3.1. Clinicopathologic Characteristics

Twenty patients with early-stage breast cancer were recruited between 2013 and 2017. The median age of breast cancer patients was 49 years (IQR: 44–58); 55% were premenopausal; and the median body mass index (BMI) was 28 kg/m^2^ (IQR: 22.5–30). There were no reported local or distant recurrences after a mean follow-up time of 62 ± 11 months. Six women reported adverse effects 2 weeks after the procedure primarily due to bruising and tenderness. In 4 of the patients, this was classified to be of grade 3 severity [13]. In some cases, it was hard to differentiate whether the adverse effects were due to the rFNA versus surgery.

### 3.2. rFNA Methylation in Tumors

**Figure 1:**
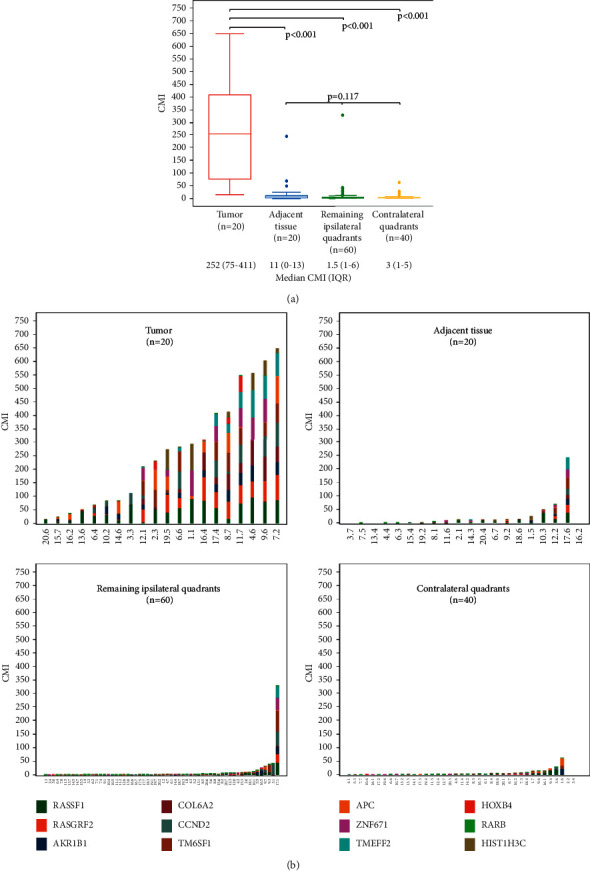
(a) Cumulative methylation index (CMI) based on rFNA samples taken from either tumor, adjacent normal tissue, or various quadrants of the breast. (b) Cumulative methylation index (CMI) by location within the breast and individual genes.

Demographics and tumor characteristics by tumor CMI are shown in Table 1. Methylation was detected in rFNA from all tumors. The median tumor CMI was not significantly different by age, race, menopausal status, BMI, or other comorbidities (Table 1). CMI was significantly higher in women with invasive ductal carcinoma lacking an associated in situ component (*p*=0.05), tumors with higher Ki67 proliferation (*p* < 0.01), and larger tumor size (*p* < 0.04). CMI was significantly higher in tumor samples (CMI = 252; IQR 75–411) compared to the adjacent normal (CMI = 11; IQR 0–13) (*p* < 0.001), as shown by the box plots in Figure 1(a). Of note, in 20% of cases, there was no detectable methylation in the adjacent tissue.

### 3.3. rFNA Methylation in Adjacent Normal and Unaffected Quadrants

Median CMI was not significantly different between adjacent tissue and remaining ipsilateral (CMI = 1.5, IQR 1–6) and contralateral (CMI = 3, IQR 1–5) quadrants sampled (*p*=0.117, Figure 1(a)). Gene-specific methylation for each rFNA sample is shown in the bar graph in Figure 1(b) and the actual numbers reported in Supplementary Table S2. The median methylation level was highest for the *RASGRF2* gene in tumors (46.5, IQR 15–76). Methylation in the *RARß, HIST1H3C,* and *ZNF671* genes were not detected in any sample.

### 3.4. Histologic Review of Adjacent Tissue and Remaining Quadrants

Comprehensive histologic review of H&E slides from the surgically excised tissue adjacent in all 20 patients and away from the primary tumor mass in 15 patients revealed cancer or premalignant lesions in 19 sections from 13 patients, consisting of invasive cancer in 4 sections (2 patients) and premalignant lesions in 15 sections (11 patients). The 15 premalignant lesions included 6 sections with ductal carcinoma in situ (DCIS) alone; 2 with DCIS and lobular carcinoma in situ (LCIS); 1 with DCIS and atypical ductal hyperplasia (ADH); 1 with DCIS, ADH, and atypical lobular hyperplasia (ALH); 3 with ADH alone; and 3 with ALH alone. Ten of the premalignant lesions were identified in adjacent normal tissue, 5 in ipsilateral unaffected quadrants, and none in contralateral quadrants. The mean tumor size of all 15 premalignant lesions was 7.8 mm (SD: 6.6). There was no significant difference in median CMI from aspirates of adjacent tissue in which premalignant lesions were (CMI = 10, IQR: 1–13) and were not detected (CMI = 11, IQR: 1–18). Similarly, there was no significant difference in the median CMI from aspirates of quadrants with (CMI = 1, IQR: 0–7) and without (CMI = 2, IQR: 1–5) lesions.

Supplementary Figure S3(a) displays box plots of CMI from rFNA collected from adjacent tissue or unaffected quadrants by categories based on the type of incidental breast lesion identified on pathology review of breast tissue. Cases with multiple premalignant histology were categorized based on the most advanced lesion, resulting in 10 DCIS and 5 ADH or ALH lesions. Supplementary Figure S3(b) shows a bar graph of the gene-specific methylation levels for each of the samples with the actual numbers reported in Supplementary Table S3(c). Eleven (73%) of the 15 premalignant lesions identified in grossly normal tissues had sufficient lesion for CMI to be evaluated on the FFPE sections, using the same 12-gene panel (Table 2). The 11 lesions consist of DCIS (*n* = 8) and ALH (*n* = 3). The mean CMI for rFNA samples collected from within the same quadrant of the 11 lesions was much lower (CMI = 8.2, SD = 14.3) than the mean CMI of the FFPE lesions (CMI = 70, SD = 96.3).

### 3.5. Family History of Breast/Ovarian Cancer

Median tumor CMI was lower in patients with at least one first degree relative with a history of breast/ovarian cancer compared to those with no family history (*p* = 0.05) as shown in Table 1. Furthermore, median CMI levels were significantly elevated in aspirates from adjacent normal tissue compared to those from the ipsilateral (*p* = 0.012) and contralateral remaining quadrants (*p* = 0.029) in women with a family history of breast/ovarian cancer (Supplementary Figure S4(a)). A similar pattern was not observed in women without a family history of breast/ovarian cancer (Supplementary Figure S4(b)). Of the 20 study participants with a family history, 9 had genetic testing prior to surgery; 1 was identified as having a pathogenic variant in BRCA2 and one had a pathogenic variant in ATM.

## 4. Discussion

To our knowledge, this is the first study to systematically quantify the presence of tumor-associated methylation markers using quadrant-based rFNA sampling. We hypothesized, based on prior tissue-based studies demonstrating a field effect [5, 19, 20], that methylation was a diffuse phenomenon during the late stages of carcinogenesis and therefore rFNA could be used to detect premalignant changes in breast tissue. In our study, rFNA consistently detected methylation in all tumor samples at high levels but not in regions in which methylated premalignant lesions were present. This could be due to the small size of the lesion, the distance of the aspirate collection from the lesion, and fine needle or assay sensitivity.

The specific 12-gene panel utilized in this study was chosen because the genes are known to be frequently methylated in at least one breast cancer subtypes [10]. The *APC*, *CCND2*, *RASGRF2,* and *TMEFF2* genes are frequently methylated in estrogen receptor-positive/progesterone receptor-positive (ER+/PR+) tumors. *APC*, *CCND2*, and *RASGRF2* genes are also frequently methylated in tumors overexpressing human epidermal growth factor receptor 2 (HER2+), and *ZNF671* is more frequently hypermethylated in triple-negative carcinomas [12].

We observed that methylation was lower in rFNA samples from the tumors of women with at least one first degree relative with breast cancer compared to those without a family history, and at higher levels in adjacent tissue. Our results are consistent with two prior studies that have reported lower levels of methylation in breast tumor tissue from *BRCA1* mutation carriers compared to noncarriers [21, 22]. A third study reported an association between lower levels of DNA methylation of *LINE-1* and *Sat2* and a more extensive family history of breast cancer in circulating white blood cells of 333 high-risk women [23]. We did not have sufficient samples from *BRCA1* mutation carriers to specifically evaluate this.

The strengths of this study include its novel design, the evaluation of a 12-gene candidate tumor-associated methylation panel, and thorough histological examination of all tissue blocks removed. A limitation of the study includes the evaluation of only a subset of all genes altered in breast tumors which is likely to reduce the chance of detecting methylation by rFNA. However, more extensive gene testing would not have increased the detection of the 12 gene panel from rFNA that were demonstrated to be present in tissue and not rFNA aspirates from the same region.

No gap here Troester et al. recently conducted a more extensive methylome analysis on 48 adjacent tissue samples and found that 15% had evidence of tumor-associated methylated genes [24].

## 5. Conclusion

The performance of a methylation panel based on rFNA sampling of adjacent tissue and quadrants of the breast to detect premalignant tissue was poor and should not be used to evaluate cancer risk.

## Figures and Tables

**Table 1:** Median tumor CMI by selected patient characteristics.

	*N* = 20 (%)	Median CMI (25^th^–75^th^ percentile)	*p* value^*∗*^
Age at surgery, years			
<50	11 (55)	111 (36–293)	0.10
≥50	9 (45)	309 (209–547)	

Race			
White	14 (70)	160 (67–408)	0.38
Black	2 (10)	433 (309–557)	
Other	4 (20)	278 (145–348)	

Menopause status			
Premenopausal	11 (55)	111 (282–649)	0.31
Postmenopausal	8 (40)	301 (179–478)	
Uncertain	1 (5)	414 (414–414)	

BMI category			
Normal (18.5–24.9 kg/m^2^)	7 (35)	83 (36–282)	0.12
Overweight/obese (≥25 kg/m^2^)	13 (65)	293 (111–414)	

Smoking status			
Never	11 (55)	111 (51–282)	0.16
Former	7 (35)	408 (83–604)	
Current	2 (10)	420 (293–547)	

No of comorbidities^‡^			
0	16 (80)	220 (59–353)	0.26
1 or more	4 (20)	359 (197–483)	

1^st^ degree family history of breast/ovarian cancer			
No	11 (55)	309 (111–547)	0.05
Yes	9 (45)	83 (36–230)	

Histologic type			
IDC	8 (40)	420 (179–581)	0.05
IDC and DCIS	12 (60)	160 (52–296)	

Tumor size, cm			
Median (IQR)	20 (100)	2.0 (1.7–2.3)	0.04
<2	8 (40)	75.5 (44–171)	
≥2	12 (60)	301 (242–481)	

TNM stage			
T1N0	8 (40)	157 (44–284)	0.69
T1N1	3 (15)	111 (67–604)	
T2N0	5 (25)	309 (282–408)	
T2N1	4 (20)	312 (146–481)	

Elston grade			
1	5 (25)	83 (67–84)	0.06
2	9 (45)	274 (36–408)	
3	6 (30)	362 (293–547)	

Type of surgery			
Lumpectomy	5 (25)	309 (84–408)	0.98
Unilateral mastectomy	6 (30)	193 (83–414)	
Bilateral mastectomy	9 (45)	230 (67–293)	

ER/PR status			
ER-positive/PR-positive	15 (75)	209 (51–408)	0.49
ER-positive/PR-negative	4 (20)	394 (157–581)	
ER-negative/PR-positive	0 (0)	—	
ER-negative/PR-negative	1 (5)	293 (293–293)	

HER2 receptor status			
Positive	6 (30)	341 (230–547)	0.19
Negative	14 (70)	147 (51–309)	

Ki67 expression, %			
<10	7 (35)	83 (51–209)	<0.01
10–30	7 (35)	230 (23–309)	
>30	6 (30)	552 (414–604)	

Molecular subtype			
Luminal A	9 (45)	67 (36–84)	<0.01
Luminal B	10 (50)	411 (274–557)	
Triple negative	1 (5)	293 (293–293)	

^*∗*^Based on Kruskal–Wallis test for categorical variables and Mann–Whitney test for continuous variables. ^‡^Comorbidities include congestive heart failure, diabetes, hypertension, and obstructive sleep apnea. BMI, body mass index; CMI, cumulative methylation index; ER, estrogen receptor; HER2, human epidermal growth factor 2; IQR, interquartile range; PR, progesterone receptor; SD, standard deviation; TNM, tumor, node, and metastasis. Luminal A = ER/PR positive, HER2 negative, and Ki67 < 14. Luminal B = ER/PR positive, HER2 negative, and Ki67 ≥ 14 or ER/PR positive and HER2 positive.

**Table 2:** Comparison of CMI from 11 incidental tissue lesions to CMI from random fine needle aspirates (rFNAs) collected from the same region or quadrant.

Histology	Size (cm)	Location	CMI of tissue lesions	CMI rFNA from same area/quadrant as lesions
ALH	0.3	Adjacent tissue	4.8	11.8
DCIS	1.0	Adjacent tissue	340.6	0.3
DCIS	2.0	Adjacent tissue	129.0	4.4
DCIS	0.4	Adjacent tissue	13.6	12.6
ALH	0.2	Ipsilateral unaffected	35.5	0.7
DCIS	0.4	Adjacent tissue	44.1	48.7
ALH	0.3	Adjacent tissue	38.0	10.0
DCIS	0.5	Adjacent tissue	14.3	0.0
DCIS	0.3	Ipsilateral unaffected	21.0	0.4
DCIS	2.0	Adjacent tissue	73.4	1.4
DCIS	1.0	Adjacent tissue	56.2	0.0

ALH, atypical lobular hyperplasia; CMI, cumulative methylation index; DCIS, ductal carcinoma in situ; rFNA, random fine needle aspiration. ^*∗*^When there was > one lesion (i.e., DCIS/ADH), samples were categorized based on the more advanced lesion.

## Data Availability

The data that support the findings of this study are available on request from the senior author, (KV). The data are not publicly available due to restrictions with them containing information that could compromise research participant privacy.

## Supplementary Materials

Supplementary Materials Supplementary Figure S1: schema for methylation study (*N* = 20 Patients). Supplementary Table S2: gene-specific methylation based on rFNA samples taken from either tumor, adjacent normal tissue, or remaining quadrants of the breast. Supplementary Figure S3(a): cumulative methylation index (CMI) of rFNA samples from adjacent tissue or quadrants where incidental (nongrossly evident) malignant or premalignant breast lesions were identified based on pathology review. Supplementary Figure S3(b): gene-specific methylation of rFNA samples from adjacent tissue or quadrants where incidental (nongrossly evident) malignant or premalignant breast lesions were identified based on pathology review. Supplementary Table S3(c): gene-specific methylation of unaffected tissue based on pathology review of adjacent normal tissue and remaining quadrants of the breast. Supplementary Figure S4(a) and 4(b): cumulative methylation index (CMI) of rFNA samples within the breast in women with a family history of breast and/or ovarian cancer (Figure 4(a)) and women with no family history of breast and/or ovarian cancer (Figure 4(b)).
