# Correction to “Evaluating DNA Methylation in Random Fine Needle Aspirates from the Breast to Inform Cancer Risk”

**DOI:** 10.1155/tbj/9873020

**Published:** 2025-11-15

K. Visvanathan, A. Cimino-Mathews, M. J. Fackler, et al., “Evaluating DNA Methylation in Random Fine Needle Aspirates from the Breast to Inform Cancer Risk,” *The Breast Journal* 2022 (2022): 9533461, https://doi.org/10.1155/2022/9533461.

In the article titled “Evaluating DNA Methylation in Random Fine Needle Aspirates from the Breast to Inform Cancer Risk,” there was an error in the Conflicts of Interest section. The corrected section appears below:

**Conflicts of Interest**

Kala Visvanathan reports funding from Cepheid and nonfinancial support from Optra Health Inc. Mary Jo Fackler reports grants from Cepheid and served as a Cepheid Consultant from February 2015 to February 2020. Mary Jo Fackler received license royalty fees from Cepheid from December 2014 through July 2021 for use of JHU cMethDNA and QM-MSP assays.

Saraswati Sukumar was awarded a grant from Cepheid and Kala Visvanathan and Mary Jo Fackler received support on it. Saraswati Sukumar served as Cepheid consultant from February 2015 through February 2020 and received license royalty fees from Cepheid (December 2014 through 2021) for use of JHU cMethDNA and QM-MSP patents. Mary Jo Fackler and Saraswati Sukumar are co-inventors of the cMethDNA assay, US patent US10450609B2. The JH Office of Policy is managing the COI for Saraswati Sukumar and Mary Jo Fackler. Vered Stearns received research grants to Johns Hopkins from Abbvie, Biocept, Novartis, Pfizer, Puma Biotechnology, and QUE. She served on the Oncology Advisory board for Novartis in 2021 and was Chair of a Data Safety Monitoring Board for AstraZeneca. She received nonfinancial support from Foundation Medicine for study assays.

We apologize for this error.

