# Supplementary material for: Evaluating DNA Methylation in Random Fine Needle Aspirates from the Breast to Inform Cancer Risk
Source: Breast J. 2022 Aug 11;2022:9533461. doi: 10.1155/2022/9533461 (PMC11401740; doi:10.1155/2022/9533461)
Supplement: Supplementary Materials — Supplementary Figure S1: schema for methylation study (N = 20 Patients). Supplementary Table S2: gene-specific methylation based on rFNA samples taken from either tumor, adjacent normal tissue, or remaining quadrants of the breast. Supplementary Figure S3(a): cumulative methylation index (CMI) of rFNA samples from adjacent tissue or quadrants where incidental (nongrossly evident) malignant or premalignant breast lesions were identified based on pathology review. Supplementary Figure S3(b): gene-specific methylation of rFNA samples from adjacent tissue or quadrants where incidental (nongrossly evident) malignant or premalignant breast lesions were identified based on pathology review. Supplementary Table S3(c): gene-specific methylation of unaffected tissue based on pathology review of adjacent normal tissue and remaining quadrants of the breast. Supplementary Figure S4(a) and 4(b): cumulative methylation index (CMI) of rFNA samples within the breast in women with a family history of breast and/or ovarian cancer (Figure 4(a)) and women with no family history of breast and/or ovarian cancer (Figure 4(b)). [file 9533461.f1.zip › Supplementary Table S2_030722.pdf]

Supplementary Table S2. Gene-specific methylation based on rFNA samples taken from either tumor, adjacent normal tissue, or remaining quadrants of the breast

Median (IQR)	Tumor N= 20	Adjacent tissue N=20	Ipsilateral quadrants N=60	Contralateral quadrants N =40	p value
**RASSF1**	46.5 (15-76)	1 (0-11)	0 (0-0)	0 (0-1)	<0.001
**RASGRF2**	24 (0-65)	0 (0-1)	0 (0-0)	0 (0-0)	<0.001
**AKR1B1**	13 (0-30)	0 (0-4)	0 (0-0)	0 (0-0)	<0.001
**COL6A2**	8 (0-47)	0 (0-0)	0 (0-0)	0 (0-0)	<0.001
**CCND2**	6 (0.5-52)	0 (0-1)	0 (0-1)	0 (0-1)	0.002
**TM6SF1**	6 (0-65)	0 (0-0)	0 (0-0)	0 (0-0)	0.012
**APC**	0.5 (0-44)	0 (0-0)	0 (0-0)	0 (0-0)	0.033
**ZNF671**	0 (0-53)	0 (0-2)	0 (0-0)	0 (0-0)	0.287
**TMEFF2**	0 (0-40)	0 (0-0)	0 (0-0)	0 (0-0)	0.022
**HOXB4**	0 (0-0.5)	0 (0-0)	0 (0-0)	0 (0-0)	0.400
**RARBeta**	0 (0-0)	0 (0-0)	0 (0-0)	0 (0-0)	0.895
**HIST1H3C**	0 (0-0)	0 (0-0)	0 (0-0)	0 (0-0)	0.319
