# Supplementary material for: Evaluating DNA Methylation in Random Fine Needle Aspirates from the Breast to Inform Cancer Risk
Source: Breast J. 2022 Aug 11;2022:9533461. doi: 10.1155/2022/9533461 (PMC11401740; doi:10.1155/2022/9533461)
Supplement: Supplementary Materials — Supplementary Figure S1: schema for methylation study (N = 20 Patients). Supplementary Table S2: gene-specific methylation based on rFNA samples taken from either tumor, adjacent normal tissue, or remaining quadrants of the breast. Supplementary Figure S3(a): cumulative methylation index (CMI) of rFNA samples from adjacent tissue or quadrants where incidental (nongrossly evident) malignant or premalignant breast lesions were identified based on pathology review. Supplementary Figure S3(b): gene-specific methylation of rFNA samples from adjacent tissue or quadrants where incidental (nongrossly evident) malignant or premalignant breast lesions were identified based on pathology review. Supplementary Table S3(c): gene-specific methylation of unaffected tissue based on pathology review of adjacent normal tissue and remaining quadrants of the breast. Supplementary Figure S4(a) and 4(b): cumulative methylation index (CMI) of rFNA samples within the breast in women with a family history of breast and/or ovarian cancer (Figure 4(a)) and women with no family history of breast and/or ovarian cancer (Figure 4(b)). [file 9533461.f1.zip › Supplementary Table S3C_030722.pdf]

Supplementary Table S3C. Gene-specific methylation of unaffected tissue based on pathology review of adjacent normal tissue and remaining quadrants of the breast

Median (IQR)	Tumor N=4	DCIS N=10	ADH/ALH N=5	Normal N=55	p value
**RASSF1**	0 (0-7)	0.5 (0-1)	0 (0-1)	0 (0-1)	0.973
**RASGRF2**	0 (0-6)	0 (0-0)	0 (0-0)	0 (0-0)	0.713
**AKR1B1**	6 (0-3)	0 (0-0)	0 (0-0)	0 (0-0)	0.482
**COL6A2**	0 (0-0)	0 (0-0)	0 (0-0)	0 (0-0)	0.312
**CCND2**	0 (0-2)	0 (0-0)	0 (0-1)	0 (0-1)	0.311
**TM6SF1**	0 (0-11)	0 (0-0)	0 (0-0)	0 (0-0)	0.397
**APC**	0 (0-32)	0 (0-0)	0 (0-0)	0 (0-0)	0.485
**ZNF671**	6 (3-8)	0 (0-0)	0 (0-0)	0 (0-0)	0.045
**TMEFF2**	0 (0-0)	0 (0-0)	0 (0-0)	0 (0-0)	0.767
**HOXB4**	0 (0-0)	0 (0-0)	0 (0-0)	0 (0-0)	0.863
**RARB**	0 (0-1)	0 (0-0)	0 (0-0)	0 (0-0)	0.837
**HIST1H3C**	0 (0-0)	0 (0-0)	0 (0-0)	0 (0-0)	0.884

*When there was > one lesion (i.e. DCIS/ADH) samples were categorized based on the more advanced lesion

DCIS = ductal carcinoma in situ, ADH = atypical ductal hyperplasia, ALH = atypical lobular hyperplasia
