# Supplementary material for: Evaluating DNA Methylation in Random Fine Needle Aspirates from the Breast to Inform Cancer Risk
Source: Breast J. 2022 Aug 11;2022:9533461. doi: 10.1155/2022/9533461 (PMC11401740; doi:10.1155/2022/9533461)
Supplement: Supplementary Materials — Supplementary Figure S1: schema for methylation study (N = 20 Patients). Supplementary Table S2: gene-specific methylation based on rFNA samples taken from either tumor, adjacent normal tissue, or remaining quadrants of the breast. Supplementary Figure S3(a): cumulative methylation index (CMI) of rFNA samples from adjacent tissue or quadrants where incidental (nongrossly evident) malignant or premalignant breast lesions were identified based on pathology review. Supplementary Figure S3(b): gene-specific methylation of rFNA samples from adjacent tissue or quadrants where incidental (nongrossly evident) malignant or premalignant breast lesions were identified based on pathology review. Supplementary Table S3(c): gene-specific methylation of unaffected tissue based on pathology review of adjacent normal tissue and remaining quadrants of the breast. Supplementary Figure S4(a) and 4(b): cumulative methylation index (CMI) of rFNA samples within the breast in women with a family history of breast and/or ovarian cancer (Figure 4(a)) and women with no family history of breast and/or ovarian cancer (Figure 4(b)). [file 9533461.f1.zip › Supplement_Tables_S2_071422.docx]

**Supplementary Table S2.** Gene-specific methylation based on rFNA samples taken from either tumor, adjacent normal tissue, or remaining quadrants of the breast

| **Median (IQR)** | **Tumor**  **N= 20** | **Adjacent tissue**  **N=20** | **Ipsilateral quadrants**  **N=60** | **Contralateral quadrants**  **N =40** | **p value** |
| --- | --- | --- | --- | --- | --- |
| **RASSF1** |  |  |  |  |  |
|  | 46.5 (15-76) | 1 (0-11) | 0 (0-0) | 0 (0-1) | <0.001 |
| **RASGRF2** |  |  |  |  |  |
|  | 24 (0-65) | 0 (0-1) | 0 (0-0) | 0 (0-0) | <0.001 |
| **AKR1B1** |  |  |  |  |  |
|  | 13 (0-30) | 0 (0-4) | 0 (0-0) | 0 (0-0) | <0.001 |
| **COL6A2** |  |  |  |  |  |
|  | 8 (0-47) | 0 (0-0) | 0 (0-0) | 0 (0-0) | <0.001 |
| **CCND2** |  |  |  |  |  |
|  | 6 (0.5-52) | 0 (0-1) | 0 (0-1) | 0 (0-1) | 0.002 |
| **TM6SF1** |  |  |  |  |  |
|  | 6 (0-65) | 0 (0-0) | 0 (0-0) | 0 (0-0) | 0.012 |
| **APC** |  |  |  |  |  |
|  | 0.5 (0-44) | 0 (0-0) | 0 (0-0) | 0 (0-0) | 0.033 |
| **ZNF671** |  |  |  |  |  |
|  | 0 (0-53) | 0 (0-2) | 0 (0-0) | 0 (0-0) | 0.287 |
| **TMEFF2** |  |  |  |  |  |
|  | 0 (0-40) | 0 (0-0) | 0 (0-0) | 0 (0-0) | 0.022 |
| **HOXB4** |  |  |  |  |  |
|  | 0 (0-0.5) | 0 (0-0) | 0 (0-0) | 0 (0-0) | 0.400 |
| **RARBeta** |  |  |  |  |  |
|  | 0 (0-0) | 0 (0-0) | 0 (0-0) | 0 (0-0) | 0.895 |
| **HIST1H3C** |  |  |  |  |  |
|  | 0 (0-0) | 0 (0-0) | 0 (0-0) | 0 (0-0) | 0.319 |

DCIS= ductal carcinoma in situ, ADH = atypical ductal hyperplasia, ALH atypical lobular hyperplasia
