# Supplementary material for: Evaluating DNA Methylation in Random Fine Needle Aspirates from the Breast to Inform Cancer Risk
Source: Breast J. 2022 Aug 11;2022:9533461. doi: 10.1155/2022/9533461 (PMC11401740; doi:10.1155/2022/9533461)
Supplement: Supplementary Materials — Supplementary Figure S1: schema for methylation study (N = 20 Patients). Supplementary Table S2: gene-specific methylation based on rFNA samples taken from either tumor, adjacent normal tissue, or remaining quadrants of the breast. Supplementary Figure S3(a): cumulative methylation index (CMI) of rFNA samples from adjacent tissue or quadrants where incidental (nongrossly evident) malignant or premalignant breast lesions were identified based on pathology review. Supplementary Figure S3(b): gene-specific methylation of rFNA samples from adjacent tissue or quadrants where incidental (nongrossly evident) malignant or premalignant breast lesions were identified based on pathology review. Supplementary Table S3(c): gene-specific methylation of unaffected tissue based on pathology review of adjacent normal tissue and remaining quadrants of the breast. Supplementary Figure S4(a) and 4(b): cumulative methylation index (CMI) of rFNA samples within the breast in women with a family history of breast and/or ovarian cancer (Figure 4(a)) and women with no family history of breast and/or ovarian cancer (Figure 4(b)). [file 9533461.f1.zip › Supplement_Tables_S3C_07140722.docx]

**Supplementary Table S3C.** Gene-specific methylation of unaffected tissue based on pathology review of adjacent normal tissue and remaining quadrants of the breast

| **Median (IQR)** | **Tumor**  **N=4** | **DCIS**  **N=10** | **ADH/ALH**  **N=5** | **Normal**  **N=55** | **p value** |
| --- | --- | --- | --- | --- | --- |
| **RASSF1** |  |  |  |  |  |
|  | 0 (0-7) | 0.5 (0-1) | 0 (0-1) | 0 (0-1) | 0.973 |
| **RASGRF2** |  |  |  |  |  |
|  | 0 (0-6) | 0 (0-0) | 0 (0-0) | 0 (0-0) | 0.713 |
| **AKR1B1** |  |  |  |  |  |
|  | 6 (0-3) | 0 (0-0) | 0 (0-0) | 0 (0-0) | 0.482 |
| **COL6A2** |  |  |  |  |  |
|  | 0 (0-0) | 0 (0-0) | 0 (0-0) | 0 (0-0) | 0.312 |
| **CCND2** |  |  |  |  |  |
|  | 0 (0-2) | 0 (0-0) | 0 (0-1) | 0 (0-1) | 0.311 |
| **TM6SF1** |  |  |  |  |  |
|  | 0 (0-11) | 0 (0-0) | 0 (0-0) | 0 (0-0) | 0.397 |
| **APC** |  |  |  |  |  |
|  | 0 (0-32) | 0 (0-0) | 0 (0-0) | 0 (0-0) | 0.485 |
| **ZNF671** |  |  |  |  |  |
|  | 6 (3-8) | 0 (0-0) | 0 (0-0) | 0 (0-0) | 0.045 |
| **TMEFF2** |  |  |  |  |  |
|  | 0 (0-0) | 0 (0-0) | 0 (0-0) | 0 (0-0) | 0.767 |
| **HOXB4** |  |  |  |  |  |
|  | 0 (0-0) | 0 (0-0) | 0 (0-0) | 0 (0-0) | 0.863 |
| **RARB** |  |  |  |  |  |
|  | 0 (0-1) | 0 (0-0) | 0 (0-0) | 0 (0-0) | 0.837 |
| **HIST1H3C** |  |  |  |  |  |
|  | 0 (0-0) | 0 (0-0) | 0 (0-0) | 0 (0-0) | 0.884 |

*When there was > one lesion (i.e. DCIS/ADH) samples were categorized based on the more advanced lesion

DCIS = ductal carcinoma in situ, ADH = atypical ductal hyperplasia, ALH = atypical lobular hyperplasia
